# Introgressive Hybridization between Anciently Diverged Lineages of *Silene* (Caryophyllaceae)

**DOI:** 10.1371/journal.pone.0067729

**Published:** 2013-07-08

**Authors:** Anna Petri, Bernard E. Pfeil, Bengt Oxelman

**Affiliations:** Department of Biological and Environmental Sciences, University of Gothenburg, Gothenburg, Sweden; Virginia Tech Virginia, United States of America

## Abstract

Hybridization has played a major role during the evolution of angiosperms, mediating both gene flow between already distinct species and the formation of new species. Newly formed hybrids between distantly related taxa are often sterile. For this reason, interspecific crosses resulting in fertile hybrids have rarely been described to take place after more than a few million years after divergence. We describe here the traces of a reproductively successful hybrid between two ancestral species of *Silene*, diverged for about six million years prior to hybridization. No extant hybrids between the two parental lineages are currently known, but introgression of the RNA polymerase gene *NRPA2* provides clear evidence of a temporary and fertile hybrid. Parsimony reconciliation between gene trees and the species tree, as well as consideration of clade ages, help exclude gene paralogy and lineage sorting as alternative hypotheses. This may represent one of the most extreme cases of divergence between species prior to introgressive hybridization discovered yet, notably at a homoploid level. Although species boundaries are generally believed to be stable after millions of years of divergence, we believe that this finding may indicate that gene flow between distantly related species is merely largely undetected at present.

## Introduction

Hybridization and introgression is frequently observed in nature, in particular among plant species. The negative correlation between documented cross-species interactions and genetic divergence (e.g., [1], [2]) reflects the fact that reproductive incompatibility generally increases with time after divergence [3]. Despite this, introgression of nuclear genes has in several cases been found to have occurred after millions of years of divergence, even where no intermediate hybrids are known (e.g., [4], [5]). The case of allele-sharing between the two grass genera *Festuca* and *Poa*, presented by Ghatnekar and Vallenback [6], [7], [8], appears exceptional, and may even be the result of horizontal gene transfer.

In this paper, we present a case of strong conflict between the species phylogeny of the genus *Silene* and the phylogeny of the low copy nuclear RNA polymerase gene *NRPA2* [9]. *Silene* is a large and diverse genus which, based on phylogenetic studies [10], [11], [12], [13], [14], [15], [16], [17] has been divided into two well supported subgenera: subgenus *Behenantha* and subgenus *Silene* (appr. 300 and 170 species, respectively; [18]). Based on chloroplast, nuclear ribosomal and low-copy nuclear DNA, Frajman et al. [14] estimated the split between the two subgenera to an age of between 9 and 13 million years (Ma). During the above listed studies of *Silene*, nuclear genes have been found to follow the expected (bifurcating) species tree well. Only a few exceptions, presumably following hybridization, have emerged. Members of section *Melandrium* (e.g., *Silene dioica* and *S. latifolia*) have been found to commonly exchange genes [19], [16], and allopolyploid species (derived from hybridization and genome doubling) are common within the Arctic/alpine section *Physolychnis* (subgenus *Behenantha*; [11], [12], [17]). Rautenberg et al. [20] detected recombination in the nuclear *XY1* gene, likely a consequence of ancient introgression between two distantly related taxa within subgenus *Behenantha*. Despite the fact that major *Silene* taxa generally appear well delimited and stable, we have found that certain members of section *Physolychnis* (subgenus *Behenantha*) exhibit a copy of the *NRPA2* gene, originating from section *Auriculatae* (subgenus *Silene*). In the following text, we will refer to the two subgenera *Behenantha* and *Silene* as clade **a** and clade **b**, respectively. Accordingly, the two divergent gene copies will be referred to as the *NRPA2 a*- and *b*-copies.

During previous studies of the involved taxa [10], [11], [12], [17], no similar pattern has been detected despite the use of three unlinked low copy nuclear genes, nuclear ribosomal DNA, and chloroplast DNA. This, and the scattered occurrence of the *b*-copy within section *Physolychnis*, leads us to believe that the few specimens containing two divergent copies are in fact not present-day hybrids between the two sections. Instead, we aim in this paper to investigate whether the presence of both an *a*-copy and a *b*-copy within the same individuals is a result of 1) introgression between members of two distinct clades, or 2) an event preceding the split of the two subgenera, i.e., gene duplication and/or lineage sorting.

## Results

### Preliminary Phylogenetic Analysis

To obtain an overview over the tribe Sileneae *NRPA2* phylogeny, a data matrix containing a large proportion of the Sileneae species (sensu Oxelman et al. [18]; excluding *Agrostemma*), was prepared. For voucher information, see Table S1. Two different recombination tests revealed no sign of recombination in the data. A nexus tree-file with a majority rule consensus *NRPA2* phylogeny from MrBayes v. 3.1.2 [21] is presented in Figure S1. Taxa marked in red were removed from the data matrix prior to *phylogenetic dating analysis* (below). All members of the diploid *Silene ajanensis* group (section *Physolychnis*) exhibit two *NRPA2 a*-copies (Figure S1). This is in agreement with the findings of Popp et al. [22], where the presence of the two *a*-copies were hypothesized to be a result of a single gene duplication. In this analysis, however, certain members of the *S. ajanensis* group (*S. villosula* 12211, *S. linnaeana* 12405, *S. samojedora* 12338, and *S. linnaeana* 12365; numbers following taxon names correspond to specimen IDs in the Sileneae database [18]) exhibit one *a-*copy and one *b*-copy. The remaining diploid *Physolychnis* taxa exhibit only one copy, with the exception of *S. viscosa*, which exhibits one *a*- and one *b*-copy. In addition, *S. sachalinensis*, a *Physolychnis* allotetraploid with *S. ajanensis* as one of its parental lineages [17], exhibits one *a*- and one *b-*copy. The *Physolychnis b*-copies form a monophyletic clade with Bayesian Posterior Probability (BPP) = 1.0, placed within (BPP = 1.0), but unresolved with respect to, the remainder of sequences sampled from section *Auriculatae*, subgenus *Silene* (Figure S1). Within this clade, the monophyly of the *S. ajanensis* group [17] is contradicted by the inclusion of *S. viscosa*, which is sister to *S. samojedora* and *S. sachalinensis* (BPP = 1.0), but no synapomorphic substitution supports this relationship. The taxonomic identities of all specimens containing one *a-* and one *b*-copy have been investigated by thorough morphological examinations. All specimens (except *S. sachalinensis* 7705) were also included in a previously published study of section *Physolychnis* [17], where two unlinked members of the *NRPA2* gene family (*RPD2a* and *RPD2b*) as well as three chloroplast markers (*matK*, *rps16*, and the *psbE-petL* spacer) were used. In that study, there was no sign of allele sharing between any of the *Physolychnis* taxa and any other member of *Silene.*

### Contamination Control

**Figure 1:**
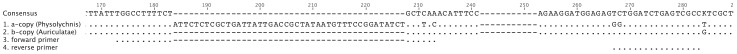
Primers designed specifically to fit the *NRPA2 b*-copy. Both primers were located in regions where all *Physolychnis* and *Auriculatae b*-copies were identical, and differing from the *Physolychnis a*-copy.

In order to exclude the possibility of sample contamination, DNA extractions of the specimens containing both an *a-* and a *b*-copy were repeated in two geographically separate labs. This procedure was successful for *Silene samojedora* 12338, *S. sachalinensis* 7033, *S. villosula* 12211 and *S. viscosa* 7705. In all these specimens, except *S. villosula* 12211, the *b*-copy was again detected in PCR and sequencing using the original *NRPA2* primers (see materials and methods), as well as with a primer pair specifically designed to amplify only the *b-*copy (Figure 1). The *b*-copy specific primers also amplified the expected *NRPA2* sequence from *S. amoena* (section *Auriculatae*), which was used as positive control, but no PCR product was obtained from specimens where the *b*-copy had previously not been found. The pairwise Jukes Cantor distances among the *Physolychnis b*-copies correspond well to the genetic distances among the *a*-copies contained within the same individuals (Table 1).

**Table 1:** Genetic distances among the *Physolychnis a*- and *b-*copies.

		1	2	3	4	5	6	7	8	9	10
**1**	S. villosula 12211 *a*	–									
**2**	S. linnaeana 12405 *a*	0.00665	–								
**3**	S. sachalinensis 6678 *a*	0.00311	0.00512	–							
**4**	S. samojedora 12338 *a*	0.00608	0.00503	0.00321	–						
**5**	S. viscosa 7705 *a*	0.02705	0.02321	0.02486	0.02483	–					
**6**	S. villosula 12211 *b*	[0.08784]	[0.08198]	[0.08430]	[0.08259]	[0.09502]	–				
**7**	S. linnaeana 12405 *b*	[0.07920]	[0.07990]	[0.07831]	[0.07720]	[0.09028]	0.00287	–			
**8**	S. sachalinensis 6678 *b*	[0.08586]	[0.08409]	[0.08645]	[0.07912]	[0.08895]	0.00428	0.00294	–		
**9**	S. samojedora 12338 *b*	[0.08784]	[0.08617]	[0.08822]	[0.08448]	[0.09424]	0.00434	0.00596	0.00579	–	
**10**	S. viscosa 7705 *b*	[0.08967]	[0.08631]	[0.08838]	[0.08445]	[0.09329]	0.00549	0.00754	0.01008	0.00874	–

Distances are calculated in PAUP* (Swofford, 2000). Numbers following taxon names correspond to specimen IDs in the *Sileneae* database [18]. Distances between the two copies are within square brackets.

Taken together, these results make it unlikely that sample contamination is the cause of the observed gene tree/species tree incongruence.

### Estimation of the Frequency of which the NRPA2 b-copy Occurs in Subgenus *Behenantha*

An estimate of the occurrence of the *NRPA2 b*-copy was obtained by amplification and sequencing with the *b*-copy specific primers (Figure 1) in all *Behenantha* species included in the *Preliminary phylogenetic analysis* (Figure S1). The expected *NRPA2* sequences was obtained from two additional taxa: *S. quadriloba* 12438, a close relative to *S. viscosa*, and *S. bungei* 14232, a *Physolychnis* allotetraploid [17]. No product was obtained from any member of subgenus *Behenantha* outside section *Physolychnis*.

### Multiple Primer Amplification

Multiple primer pairs were constructed for amplification of the *NRPA2* gene within *Silene* by the aid of two transcriptome libraries - one from *Silene uralensis* (section *Physolychnis*) and one from *S. schafta* (section *Auriculatae*). In these, only one *NRPA2* copy was found during local BLAST searches. The mean read depth of contigs matching *Arabidopsis NRPA2* was 4.2 (four non-overlapping contigs) for *S. uralensis* and 4.1 (two non-overlapping contigs) for *S. schafta*.

57 primers were constructed, amplifying seven *NRPA2* introns in 111 primer combinations (Table S2). From 654 PCR reactions on twelve *Silene* specimens (see *materials and methods*), 235 readable sequences were recovered. All sequences from *S. viscosa* and *S. sachalinensis* (except one) were polymorphic and exhibited one sequence matching the clade **a** reference sequence and one matching the clade **b** reference sequence. All representatives of the *S. ajanensis* group exhibited two sequences, but none of them matched the clade **b** reference (in accordance with Popp et al. [22], in which a single gene duplication of *NRPA2* is inferred). Surprisingly, the *b*-copy could not be detected in *S. samojedora* 12338, which beyond doubt does exhibit it (as seen from PCR amplification with the primers from Popp and Oxelman [11], as well as with the *b*-copy specific primers), even though the primers amplify the *b-*copy well from both *S. sachalinensis* and *S. viscosa*. The reason for this can only be speculated on at this point, but may be scope for further investigation. *Silene amoena* (section *Auriculatae*) exhibited two sequences, but neither of them matched the clade **a** reference. This is likely to be caused by polyploidy within clade **b**, as *S. amoena* has been reported as both diploid and tetraploid (as *S. repens* in IPCN, www.tropicos.org/name/6301554?projectid=9). The two clade **a** and clade **b** reference individuals, *S. uralensis* and *S. boryi*, exhibited occasional single nucleotide polymorphisms (SNPs), but only one base (in one sequence of *S. uralensis*) was diagnostic for the other subgenus. This is likely to be an isolated case of homoplasy. The results of the SNP scoring are presented in Table 2. Alignments with chromatograms are available upon request from the corresponding author.

**Table 2:** Results from *Multiple primer amplification.*

Specimen name	A	B	C
*S. uralensis*	16	16^a^	
*S. ajanensis* group without *b*-copy	42	42^d^	
*S. ajanensis* group with *b*-copy	35	35^a^	
*S. viscosa*	21	21^b^	18
*S. sachalinensis*	31	31	31
*S. boryi*	44		44
*S. amoena*	46		46c,d

a One sequence contains one site diagnostic to the other section.

b Three sequences contains one site each diagnostic to the other section.

c 15 sequences share one site diagnostic to the other section.

d 5 (4) sequences share one site diagnostic to the other section.

Column A) No. readable sequences, B) No. of sequences matching clade a reference (*S. uralensis*), C) No. of sequences matching clade b reference (*S. boryi*). Matches to reference sequences were determined via SNP detection.

### Phylogenetic Dating Analysis

**Figure 2:**
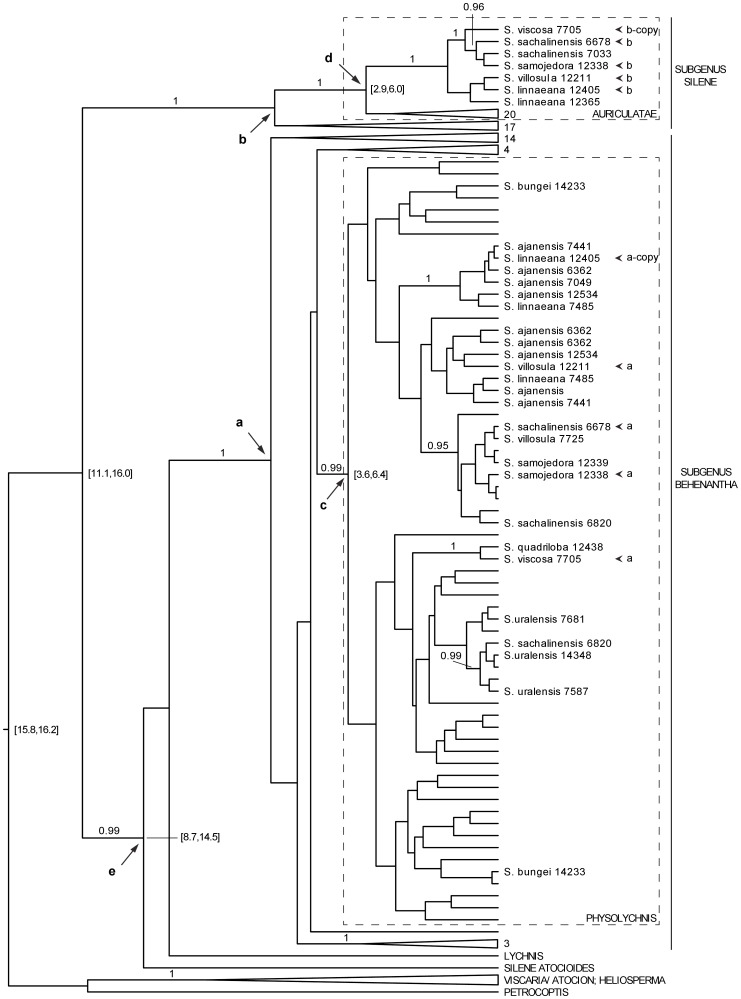
Maximum clade credibility tree from the dated *NRPA2* phylogeny. Clades are collapsed and taxon names removed if insignificant for the discussion, but within the ingroup followed by a number denoting the number of taxa retained within the clade (in Figure S2). Numbers following taxon names correspond to specimen IDs in the *Sileneae* database [18]. Bayesian posterior probabilities ≥0.95 are plotted above branches leading to clades relevant to this study. 95% HPD age intervals are indicated within square brackets opposite nodes that are relevant to the discussion. Individuals containing both an *a*- and a *b*-copy are high-lighted with arrowheads. Nodes discussed in the text are labeled **a** through **e**.

For estimation of relative divergence times of the *NRPA2* gene copies, the data matrix from *Preliminary phylogenetic analysis* (Figure S1) was pruned to contain fewer taxa. Despite the exclusion of specimens residing on long branches, the 95% HPD interval of the coefficient of variation of substitution rates among branches did not include zero, indicating that the *NRPA2* gene does not evolve in a strict clock-like manner [23]. A simplified image of the Maximum clade credibility tree from the dated phylogeny in BEAST v.1.7.1 [24] under an uncorrelated clock with rates distributed on branches according to a lognormal distribution is presented in Figure 2, where nodes and clades referred to in the text below are indicated. The complete nexus tree file is presented in Figure S2.

The two subgenera *Behenantha* and *Silene* are well supported in Figure 2 (node **a** and **b**, respectively). Since the sister relationship between the two subgenera is unresolved, their divergence age cannot be determined (but is still indicated in Figure 2; 11.1, 16.0). After exclusion of the red-marked taxa in Figure S1, section *Physolychnis* is supported with 99% BPP (node **c** in Figure 2), but the resolution within the section is poor. In accordance with *preliminary phylogenetic analysis*, section *Auriculatae* receives high support (node **d** in Figure 2). The *Physolychnis b*-copies form one well supported clade within section *Auriculatae*. In agreement with *preliminary phylogenetic analysis* (Figure S1), *S. viscosa* is sister to *S. sachalinensis* and *S. samojedora* within this clade.

### Gene Duplication/Loss

Gene duplications and subsequent losses could lead to topological incongruence between gene trees and their underlying species tree. We examined the number of duplications and losses required when duplications are minimized using GeneTree v. 1.3.0 [25]. Given the topology from *phylogenetic dating analysis* (Figures 2, S2), a duplication/loss scenario would require four independent gene duplications, including one prior to the split of the two subgenera, and 37 independent losses. The *Physolychnis b*-copy branches off close to the tip of the tree, requiring more duplications and losses in order to reconcile this phylogeny with the expected species tree, beyond just one single duplication (which would be the minimum required to explain two divergent copies within a species). A tree displaying a hypothetical duplication and loss scenario is presented in Figure S3.

## Discussion

The presence of divergent sequences within samples may be explained in several ways. We can reject contamination, lineage sorting and gene duplication, but we cannot reject an introgression scenario, as described in the following text.

### Introgression as a Model to Explain the Physolychnis *b*-copy

In the absence of gene loss, incomplete sampling, extinction, or lineage sorting, introgressive hybridization can be expected to produce a phylogenetic pattern where both gene copies retained within one species coalesce with their parental lineages at the same time. In the dated *NRPA2* phylogeny (Figure 2), this criterion is fulfilled, as the divergence time of node **d** (the starting point of divergence of the *Physolychnis b*-copies), overlaps with that of node **c** (the starting point of divergence of the *Physolychnis a*-copies). The *Physolychnis b*-copies are monophyletic (Figure 2), consistent with a scenario of a single introgression event from a member of section *Auriculatae*, to a member of section *Physolychnis*. Even though distribution patterns and ecological preferences of the included taxa are unknown for the estimated time of introgression (see below), current data do not contradict this hypothesis. Members of the *Silene ajanensis* group grow in sympatry with *S. amoena*, which is a Far Eastern member of section *Auriculatae*.

**Figure 3:**
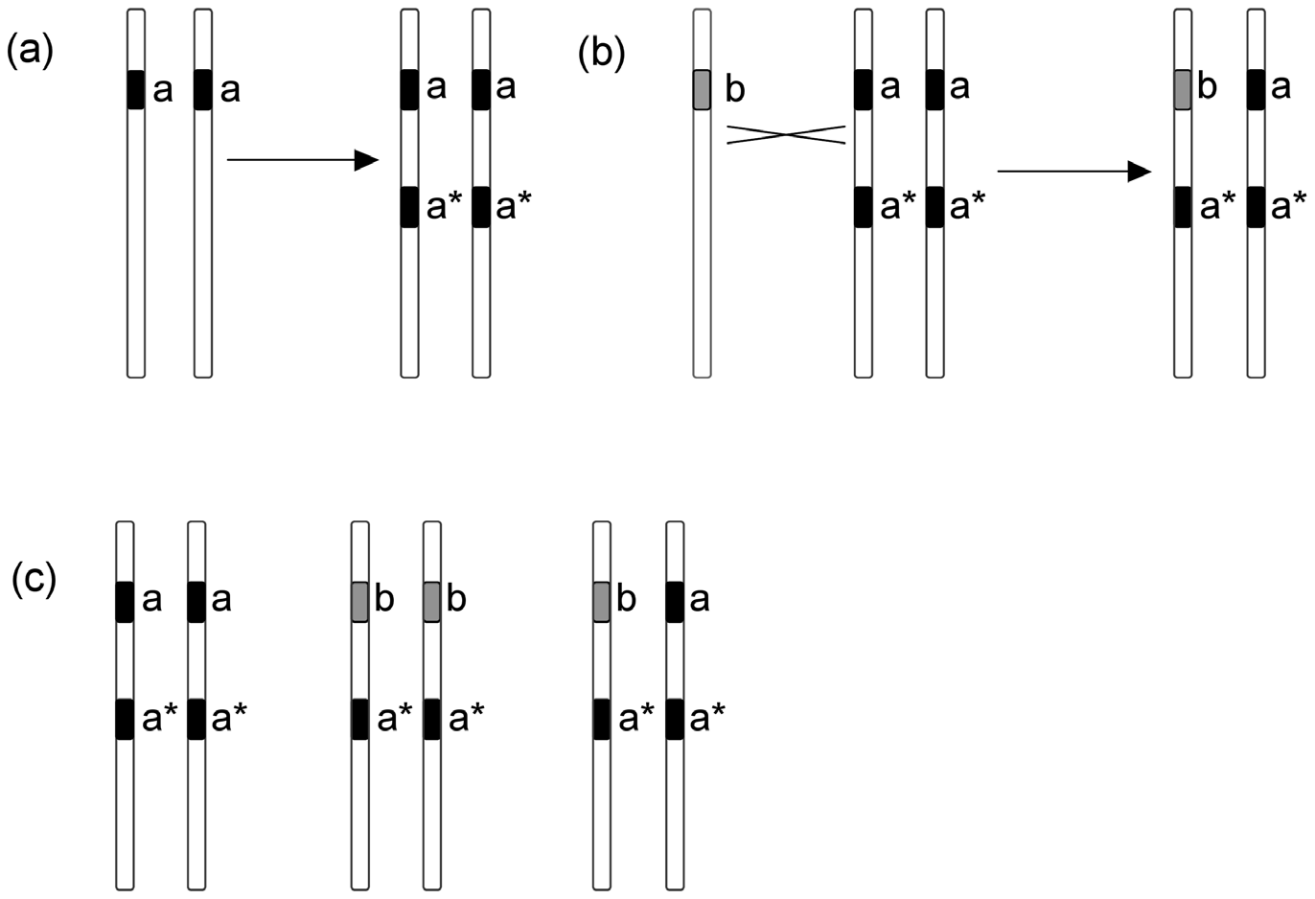
A hypothetical scenario of introgression, involving the *Silene ajanensis* group and section *Auriculatae*. a) In an ancestor of the *S. ajanensis* group (black), the *NRPA2* gene is duplicated. As a result, this group contains two monophyletic gene copies, *a* and a*. b) Mediated by a temporary hybrid, a gene copy *b* from a member of section *Auriculatae* (grey) is introgressed into the *S. ajanensis* genome. c) As a result, we can observe the following gene copy combinations in the *S. ajanensis* group: aa* aa*; ba* ba*; ba* aa*.

A hypothetical model of introgression, explaining all three *NRPA2* gene copies found within the *S. ajanensis* group, is presented in Figure 3. a) The *a*-copy (*black*) is duplicated resulting in *a* and a* copies, b) the *b*-copy (*gray*) is introgressed from section *Auriculatae*, c) as a result, the following gene copy combinations are present in the genome of the *S. ajanensis* group: aa* aa* (has been observed as the normal case within the group); ba* ba* (has potentially been observed – it cannot be determined from the recovered sequences or the phylogenetic tree whether the *a*-copy is the original or the duplicate when the *b*-copy is also present); ba* aa* (has likely been observed in *S. samojedora* 12338, in which a *b*-copy was initially found, and from which two *a*-copies were amplified during *multiple primer amplification*).

We cannot precisely establish the timing of the introgression event, but it is roughly estimated to have occurred at the time of node **d** (between 2.9 and 6.0 million years ago, Figure 2) or slightly younger if deeply coalescing alleles have survived in the descendants [26]. Given the estimated time of divergence of the subgenera (9–13 million years ago; [14]), the involved taxa had been separated for 3.0–10.1 Ma at the point of introgression, and are likely to have been well isolated. After such long time of divergence, a cross resulting in an (at least temporarily) fertile and stable hybrid is remarkable. Hybridization leaving evolutionary lasting traces after a mean of 6.4 Ma of divergence has been observed previously [27], but that appears to be an extreme example – no other studies that we are aware of have reported similar magnitudes of parental divergence. Kruckeberg [28], [29] managed to produce F1 hybrids from crosses between members from different clades stemming from the 5.7 Ma radiation at the base of *Silene* subgenus *Behenantha* [30]. Crang and Dean [31] reported seed formation from artificial crosses between *S. latifolia* and *S. antirrhina* L. (subgenus *Silene*), and also between *S. latifolia* and *Atocion armeria* (L.) Raf., which share a most recent common ancestor as old as 15 Ma [14]. In all of these cases, the F1 hybrids were sterile.

Apart from the *S. ajanensis* group, four taxa have here been shown to exhibit the *NRPA2 b*-copy: *S. sachalinensis*, *S. viscosa*, *S. bungei*, and *S. quadriloba* (the latter two by amplification with *b*-copy specific primers). The allopolyploids *S. sachalinensis* and *S. bungei* may have inherited the *b*-copy from the *S. ajanensis* group, from which they are both derived [17]. The diploid *S. viscosa* and its closest relative *S. quadriloba*, which are distinct from the *S. ajanensis* group (e.g., [16], [17]), may have acquired the *b*-copy from the *S. ajanensis* group in a secondary introgression event. Alternatively, a lineage ancestral to *Physolychnis* received the *b*-copy, in which case it has been lost in all other extant *Physolychnis* taxa. The *S. ajanensis* group was strongly supported as monophyletic in Petri and Oxelman [17] and Popp et al. [11]; using 3 and 6 unlinked genetic markers, respectively). This monophyly is here contradicted in the *Physolychnis b*-copies (Figures 2, S1, S2) by the inclusion of *S. viscosa*. However, this clade is not supported by any synapomorphic substitution and may be an inference artifact. Without further evidence of the validity of this clade, or supported resolution among the *Physolychnis a*-copy sequences, we cannot confidently reject or accept any hypothesis regarding this topological incongruence and the presence of the *NRPA2 b*-copy in *S. viscosa*.

### Paralogy is an Inadequate Explanation

The gene duplication/loss criterion in GeneTree v. 1.3.0 [25] (Figure S3) calculated that 4 duplications and 37 losses are required to explain the presence of the *Physolychnis b*-copy in the *NRPA2* gene tree. Not only is this less parsimonious than a single hybridization event, but after studying the outcome of the dating analysis, we are able to reject this hypothesis on the following grounds:

Although the lack of good calibration points within the tree makes us unable to establish absolute node ages, the relative ages provide the necessary information. A gene duplication scenario implies that there are several nodes in the reconciled gene tree that correspond to the divergence of the two subgenera (nodes **d** in Figure S3). In Figure 2, the split of the two subgenera is represented by node **d**, as well as by an unresolved node close to the base of the tree (hypothetically the unassigned node with an indicated age interval 11.1–16.0 Ma, BPP<0.95). The remainder are lacking because of implied gene losses. If node **d** in Figure 2 truly represents the divergence of the two subgenera, it must be older than all nested nodes, including the node that groups all ingroup taxa that are not part of subgenus *Silene* (nodes **e** in Figures 2, S3). However this is not the case. The 95% HPD interval of the height of node **e** is older and does not overlap the 95% HPD interval of the height of node **d** (Figure 2). This rejects the most parsimonious gene duplication scenario as inferred by our GeneTree analysis.

### Lineage Sorting is an Inadequate Explanation

Lineage sorting is also known to cause gene tree/species tree conflicts such as those we have observed here, and thus requires consideration. Similar to the scenario of paralogy, the retention of a minimum of 4 alleles over time is required to explain the *NRPA2* topology. However, node **d** must be older than node **e** under the scenario of lineage sorting, in the same way as under the scenario of gene duplications and losses, which also enables us to confidently exclude this hypothesis.

### Conclusions

The two subgenera within *Silene* have been well delimited in previous phylogenetic studies, but we have here presented a case where this does not hold true. Instead, certain members of subgenus *Behenantha* section *Physolychnis* exhibit an extra copy of the *NRPA2* gene, originating from subgenus *Silene* section *Auriculatae*. We have ruled out several alternative scenarios and are instead left with the remnant of a single and unidirectional introgression event as the explanation for our observations. This event likely took place about 6.6 Ma after the two subgenera had diverged. Even though hybridization within the genus *Silene* has been documented previously (e.g., [11], [19], [12], [17]), the formation of a fertile and stable hybrid is remarkable after such a long time of divergence. Presently, there are no documented hybrids between subgenus *Behenantha* and subgenus *Silene* that could serve as genetic ‘bridges’.

As concluded by Edmands [3], no clear cut boundary of time since species divergence ensuring complete reproductive isolation can be set for all species groups. Nonetheless, we are only aware of very few documented cases where sharing of genetic material has occurred between species with such a degree of divergence as presented here. Joly et al. [27] described a case of allopolyploidization involving *Brassicaceae* taxa with a divergence time similar to those documented here, but as the authors point out, this event represents one of the most extreme divergences between parental lineages of a detected hybrid. Increased parental divergence appears to be positively correlated with polyploid formation rather than homoploid hybrid formation [32] (but see [33]), and as we have no reason to suspect hybridization other than that at a homoploid level, our results may be even more remarkable than the allopolyploidization events documented by Joly et al. [27]. Therefore, our finding may rank as one of the most extreme examples of parental divergence leading not just to a reproductively successful hybrid, but at a homoploid level in particular.

At the present time, gene flow between species of such a level of divergence is often not expected, and indications of such may be discarded as contamination. Together with the findings of Rautenberg et al. [20] and Ghatnekar and Vallenback et al. [6], [7], [8], our finding may indicate that gene flow between distantly related taxa may in fact not be unusual, only largely undetected. We believe that as more data from low copy nuclear genes become available, more evidence for long-lasting evolutionary traces of gene flow between divergent species will be presented.

## Materials and Methods

DNA extractions, PCR amplification, sequencing, and sequence editing were done as described in Petri and Oxelman [17]. The primers used for amplification of the *NRPA2* gene were those designed by Popp and Oxelman [11]. Sequences new to this study have GenBank accession numbers KC522717-KC522820.

### Preliminary Phylogenetic Analysis

*NRPA2* sequences at the *Sileneae* database [18], excluding *Agrostemma* sequences, were downloaded and aligned in MUSCLE [34] at the EBI Web Service (http://www.ebi.ac.uk/Tools/muscle/). Manual alignment adjustment was made in Se-Al v.2.0a11 [35], resulting in an alignment containing 227 taxa and 1055 characters, of which 393 characters are parsimony informative, and 498 characters are constant. The data matrix can be downloaded from TreeBase, with Study Accession URL http://purl.org/phylo/treebase/phylows/study/TB2:S14183. The DualBrothers [36], [37] plug-in for Geneious v.5.3 [38] was used for recombination detection, using a preliminary scanning window length size of 400. In addition, a subset of 90 sequences from the *NRPA2* matrix was constructed based on the results from a preliminary phylogenetic analysis, where at least one sequence was chosen from each of the smallest clades with a posterior ≥0.98. This dataset was used for recombination detection with the GARD (Genetic Algorithm Recombination Detection) [39] web service (www.datamonkey.org). The model used for the analysis was chosen by the model test available at the Datamonkey web server. Phylogenetic analyses was performed in MrBayes v.3.1.2 [21], using a GTR+I+G model (chosen under the AIC criterion by MrModeltest v2.3 [40]). Two runs with four chains each were run 10 million generations. Convergence of the MCMC chains was confirmed in Tracer v1.5 [41]. Examination of the splits in AWTY [42] revealed that two of the runs had performed better, which is why only these were used when summarizing the phylograms (40% burnin).

### Contamination Control

New DNA extractions and amplification and sequencing of the *NRPA2* gene were done twice using fresh chemicals in two geographically separated labs (Evolutionary Biology Center, Uppsala University and Dept. of Biological and Environmental Sciences, University of Gothenburg) from each *Physolychnis* specimen where the *NRPA2 b*-copy was found during *preliminary phylogenetic analysis*, followed by *Silene amoena* 12609 (section *Auriculatae*).

One primer pair was constructed specifically to fit the *NRPA2 b-*copy (Figure 1), where the forward primer was located at an insertion site in *a*-copy sequences, and the 3′ base was located at a A/C polymorphism between the two copies. The two 3′ bases of the reverse primer were located at G/T and G/C polymorphisms between the two copies. The mis-matches toward the *a*-copy sequences alone ought to ensure specific amplification of only *b*-copy sequences [43]. The primer pair was used for PCR amplification and sequencing of *Physolychnis* specimens both in which the *b*-copy had previously been found and where it had not, as well as of *S. amoena* 12609.

From *Physolychnis* specimens which exhibit the extra gene copy, pairwise Jukes Cantor distances among the *a*- and *b*-copies were calculated in PAUP* [44].

### Estimation of the Frequency of which the NRPA2 b-copy Occurs in *Behenantha*

The *b*-copy specific primer pair (Figure 1) was used for amplification and sequencing of a total of 111 species from subgenus *Behenantha* (119 specimens). The *ITS* primers developed by Popp and Oxelman [45], known from previous studies to amplify well in *Silene*, were used as quality control for the DNA.

### Multiple Primer Amplification

To increase the chance of detecting all *NRPA2* copies present in the *Silene* genome, a set of new primers were constructed and combined in amplification and sequencing as follows:

RNA was extracted from one individual of *S. uralensis* (Rupr.) Bocquet subsp. *arctica* (Th. Fr.) Bocquet, grown from seeds collected in Svalbard, Endalen, 52 m above sea level, and cultivated in the phytotron at Tøyen, University of Oslo (Gustavsson 14, LG09-S-14-01 to LG09-S-14-10, vouchers at herbarium O), and one of *S. schafta* J.G.Gmel. ex Hohen. from the Botanical Garden in Gothenburg (voucher: Oxelman 2565, deposited at herbarium GB), using the mirVana miRNA isolation kit (Ambion) by vertis Biotechnologie AG (http://www.vertis-biotech.com). As many stages of the life-cycle as possible were used (i.e., roots, stem, old and young leaf buds, flowers, developing fruits), ensuring as complete mRNA coverage as possible. The resulting EST libraries were normalized by one cycle of denaturation and reassociation of the cDNA, resulting in N1-cDNA. Reassociated ds-cDNA was separated from the remaining ss-cDNA (normalized cDNA) by passing the mixture over a hydroxylapatite column. After hydroxylapatite chromatography, the ss-cDNA was amplified with 11 PCR cycles. The cDNAs in the size range of 500–700 bp were eluted from preparative agarose gels, tagged by species -specific barcodes, and sequenced on a half picotiter plate on a 454 GS-FLX sequencer with Titanium reagents (Roche) at the Norwegian Sequencing Center (http://www.sequencing.uio.no). Newbler v. 2.5 (Roche) was used for transcriptome assembly, and run with the “-cdna” option to assemble transcriptomes using the following settings: minimum overlap length = 40, minimum overlap identity = 90, alignment identity score = 2, and alignment difference score = –3.

The two resulting EST libraries were used as databases in TBLAST and TBLASTX searches using blast v.2.2.23+ [46], with the complete *NRPA2* sequence of *Arabidopsis* (NCBI GenBank) as query. Contigs that gave significant hits were blasted against the NCBI GenBank nucleotide database, and those that matched *NRPA2* (and no other gene of the same gene family) were used for primer construction. The complete *NRPA2* sequence and an *NRPA2* exon sequence from *Arabidopsis* (NCBI GenBank) were manually aligned to the *Silene NRPA2* transcriptome contigs in Geneious Pro v.5.0–5.1 [38]. Primers were designed in positions where amino-acids were relatively conserved between *Silene* and *Arabidopsis*, but based only on *Silene* nucleotide sequences. For forward primers, 3′ ends were set at 2^nd^ codon position, and amino acids with degenerate 1^st^ codon positions were avoided when possible. Non-degenerate 1^st^ codon positions where set as 3′ end for reverse primers. For all primers, degenerate 1^st^ codon positions were never used closer than four bases from the 3′ end. Primers were screened for self-complementarity at Eurofins MWG webservice (www.operon.com). 23 forward and 31 reverse primers were constructed, which were combined in PCR amplification such that all forward primers for each intron were once paired with each of the reverse primers for the same intron, making 111 primer combinations; one for the intron corresponding to *Arabidopsis* intron 9, two on intron 10, six on intron 11, six on intron 17, 14 on intron 23, 46 on intron 24, and 36 on intron 25. Primer sequences are listed in Table S2. PCR amplification and sequencing was performed on the following specimens:

one specimen from the *S. ajanensis* group where the b-copy was found during *preliminary phylogenetic analysis* (*S. samojedora* 12338),
three *S. ajanensis* group specimens where the b-copy was not found during *preliminary phylogenetic analysis* (*S. samojedora* 12396 and 12339, *S. ajanensis* 7049),
two *Physolychnis* specimens outside the *S. ajanensis* group (*S. uralensis* 12597, 14348)
*S. viscosa* 7705, 12449, 2498,
*S. sachalinensis* 7033,
two representatives of section *Auriculatae* (*S. boryi* 6165, *S. amoena* 12609).

After sequencing, the chromatograms were edited and aligned in Geneious Pro v.5.0–5.1 [38]. Polymorphic regions caused by length differences were pruned, and the sequences were searched for Single Nucleotide Polymorphisms (SNPs) diagnostic for the subgenera. The two diploids *S. uralensis* and *S. boryi* were used as references for the *a*-copy (subgenus *Behenantha*) and the *b*-copy (subgenus *Silene*) sequences, respectively.

### Phylogenetic Dating Analysis

From the *NRPA2* matrix used in *preliminary phylogenetic analysis*, a simpler and more clock-like dataset was obtained by removing taxa residing on long branches, as well as several taxa from small and well supported clades (based on the *preliminary phylogenetic analysis*; the taxa marked in red in Figure S1). This resulted in a data matrix containing 138 taxa and 998 characters, of which 240 characters are parsimony informative, and 569 characters are constant. The data matrix can be downloaded from TreeBase, with Study Accession URL http://purl.org/phylo/treebase/phylows/study/TB2:S14183. Phylogenetic dating was performed in BEAST v.1.7.1 [24] using the uncorrelated lognormal relaxed clock. The nucleotide substitution model was set to GTR, base frequencies estimated, site heterogeneity model Gamma (four gamma categories). The speciation tree prior was set to Yule process, root height prior distribution set to normal (with mean 16 and standard deviation 0.1; initial value 16), ucld.mean/clock.rate uniform (0,1; initial value 0.01). All other settings were left as defaults from BEAUti v.1.6.1 (available at http://beast.bio.ed.ac.uk). The prior age distribution of the root of the tree was taken from Frajman et al. [14], who estimated the age of *Sileneae* except *Agrostemma* to between 8.7 and 22 Ma. The tree obtained from *preliminary phylogenetic analysis*, with the taxa marked in red in Figure S1 removed, was transformed into a chronogram using non-parametric rate smoothing and scaled at the root to 15.8 in TreeEdit v.1.1 [47] and used as a starting tree. Four independent MCMC chains were run 100 million generations. The chains were diagnosed in Tracer v.1.5 [41], after which the run in which the splits had converged best (diagnosed in AWTY [42]) was used to calculate the maximum clade credibility tree in TreeAnnotator v1.6.1 (http://beast.bio.ed.ac.uk), using a burn-in of 30%.

### Gene Duplication/Loss

The number of gene duplications and losses required to explain the presence of the *NRPA2 b*-copy in section *Physolychnis* was calculated using GeneTree v. 1.3.0 [25]. The *Physolychnis b*-copies were removed from the *phylogenetic dating analysis* tree in order to make the species tree. In the gene tree, only the *Physolychnis b*-copy sequences were assigned to the corresponding individuals in the **a**-clade. In this way, we assessed only how many duplications and losses would be required to accommodate the *Physolychnis b*-copy without regard to any other sources of locus duplication or incomplete lineage sorting.

## Supporting Information

Figure S1 Preliminary phylogenetic analysis. Majority rule consensus tree from MrBayes v.3.1.2 [21], Nexus tree file format. Taxon names are followed by specimen ID from the *Sileneae* database [18] and GenBank accession number.
(TRE)

Figure S2 Phylogenetic dating analysis. Maximum clade credibility tree from BEAST v.1.7.1 [24], Nexus tree file format. Taxon names represent specimen and sequence ID from the *Sileneae* database [18].
(TRE)

Figure S3 Gene duplication/loss. A most parsimonious gene duplication/loss scenario calculated by GeneTree v. 1.3.0 [25]. Black squares represent gene duplications, where the small square (leading to *Behenantha b*-copeis 1 and 2) represents a duplication required to accommodate the inclusion of *Silene viscosa* in the *S. ajanensis* group (see Results and Discussion for details). Red branches in the tree represent gene losses. Node **d** and node **e** correspond to the nodes in Figure 2 and referred to in the discussion.
(PDF)

Table S1 Voucher information. Tab separated voucher information for all specimens included in the study. Specimen IDs are from the *Sileneae* database [18], and herbarium acronyms follow [48].
(CSV)

Table S2 Multiple primer amplification. List of primers used to amplify *NRPA2* during the *multiple primer amplification*. Primer names are initiated with the gene name (RPA2), followed by ‘u’ or ‘us’, depending on whether the primers were constructed from only *Silene uralensis* sequences (u) or from both *S. uralensis* and *S. schafta* (us) sequences. Then follows a number (-X-) denoting which intron (corresponding to *Arabidopsis* intron number) the primer is designed to amplify. Last the primer number and F for forward primers and R for reverse primers.
(TXT)
