# Supplementary figures and images for: Introgressive Hybridization between Anciently Diverged Lineages of Silene (Caryophyllaceae)
Source: PLoS One. 2013 Jul 8;8(7):e67729. doi: 10.1371/journal.pone.0067729 (PMC3704521; doi:10.1371/journal.pone.0067729)

Figure S3  
Gene duplication / loss scenario

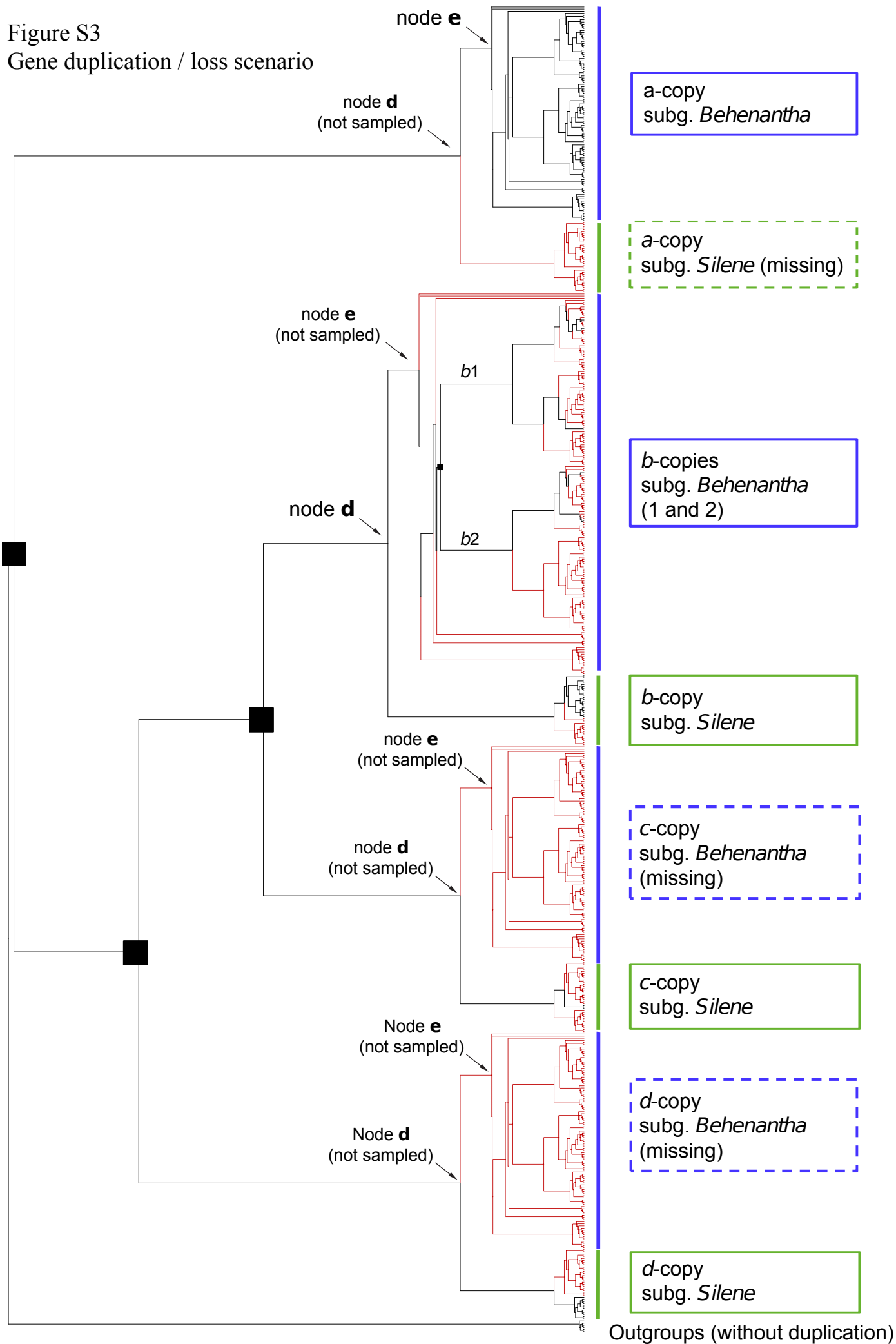

Supplement: Figure S3 — Gene duplication/loss. A most parsimonious gene duplication/loss scenario calculated by GeneTree v. 1.3.0 [25]. Black squares represent gene duplications, where the small square (leading to Behenantha b-copeis 1 and 2) represents a duplication required to accommodate the inclusion of Silene viscosa in the S. ajanensis group (see Results and Discussion for details). Red branches in the tree represent gene losses. Node d and node e correspond to the nodes in Figure 2 and referred to in the discussion. (PDF) [file pone.0067729.s003.pdf]
